# Identification of Soil Properties Associated with the Incidence of Banana Wilt Using Supervised Methods

**DOI:** 10.3390/plants11152070

**Published:** 2022-08-08

**Authors:** Barlin O. Olivares, Andrés Vega, María A. Rueda Calderón, Juan C. Rey, Deyanira Lobo, José A. Gómez, Blanca B. Landa

**Affiliations:** 1 Doctoral Program in Agricultural, Food, Forestry Engineering and Sustainable Rural Development, Rabanales Campus, University of Cordoba, Carretera Nacional IV, km 396, 14014 Cordoba, Spain; 2 Faculty of Agricultural Sciences, National University of Cordoba, Av. Haya de la Torre s/n, Cordoba 5016, Argentina; 3 Laboratorio de Genética y Genómica Aplicada, Escuela de Ciencias del Mar, Pontificia Universidad Católica de Valparaíso, Av. Universidad 330, Valparaíso 2950, Chile; 4 National Center for Agricultural Research, National Institute of Agricultural Research (INIA-CENIAP), Av. Universidad vía El Limón, Maracay 02105, Venezuela; 5 Soil Science Department, Faculty of Agronomy, Central University of Venezuela, Av. Universidad, Maracay 02105, Venezuela; 6 Institute for Sustainable Agriculture (IAS), Spanish National Research Council (CSIC), Avenida Menéndez Pidal s/n, 14004 Cordoba, Spain

**Keywords:** calcium, clay, iron, machine learning, random forest, zinc

## Abstract

Over the last few decades, a growing incidence of Banana Wilt (BW) has been detected in the banana-producing areas of the central zone of Venezuela. This disease is thought to be caused by a fungal–bacterial complex, coupled with the influence of specific soil properties. However, until now, there was no consensus on the soil characteristics associated with a high incidence of BW. The objective of this study was to identify the soil properties potentially associated with BW incidence, using supervised methods. The soil samples associated with banana plant lots in Venezuela, showing low (*n* = 29) and high (*n* = 49) incidence of BW, were collected during two consecutive years (2016 and 2017). On those soils, sixteen soil variables, including the percentage of sand, silt and clay, pH, electrical conductivity, organic matter, available contents of K, Na, Mg, Ca, Mn, Fe, Zn, Cu, S and P, were determined. The Wilcoxon test identified the occurrence of significant differences in the soil variables between the two groups of BW incidence. In addition, Orthogonal Least Squares Discriminant Analysis (OPLS-DA) and the Random Forest (RF) algorithm was applied to find soil variables capable of distinguishing banana lots showing high or low BW incidence. The OPLS-DA model showed a proper fitting of the data (R^2^Y: 0.61, *p* value < 0.01), and exhibited good predictive power (Q^2^: 0.50, *p* value < 0.01). The analysis of the Receiver Operating Characteristics (ROC) curves by RF revealed that the combination of Zn, Fe, Ca, K, Mn and Clay was able to accurately differentiate 84.1% of the banana lots with a sensitivity of 89.80% and a specificity of 72.40%. So far, this is the first study that identifies these six soil variables as possible new indicators associated with BW incidence in soils of lacustrine origin in Venezuela.

## 1. Introduction

Bananas (*Musa* spp.) represent an important crop for Venezuela’s economy, which is predominantly based on oil. During the last 20 years, banana production has undergone slight reductions, reaching 650,051 tons in 2019, with a cultivated area of around 41,708 ha, partially due to the shortage of agricultural inputs (fertilizers and agrochemicals), problems of access to foreign currency to meet domestic demand, the inadequate management of agricultural policies and the impact of drought, pests and diseases [1].

Banana Wilt (BW), also called “False Panama Disease” was first described in South Africa by Deacon et al. [2]. Although some of the *Fusarium* species have been associated with BW plants, pathogenicity tests using those strains were not successful and the etiology of BW could not be established. Both biotic and abiotic factors [3], including some physical and chemical soil characteristics and potentially pathogenic soil microorganisms [4] have been referred to as the potential causes of BW. However, BW is a disease of unknown etiology up to date, and is mainly considered as a physiological and metabolic plant disorder, whose symptoms can be easily confounded with those of Fusarium wilt, caused by *Fusarium oxysporum* f.sp. *cubense* (Foc) (Sin: *Fusarium odoratissimum*), considered one of the most destructive diseases of bananas worldwide [5,6].

In the Aragua state of Venezuela, one of the main producing areas in the country, the yields of Cavendish bananas have been decreasing since 2006 associated with the BW disease [7,8], increasing the concerns of the farmers. However, since the causal agent of this disease has not yet been properly identified, its prevention and control are difficult.

The scientific literature and the evidence in the field of Martínez et al. [7] and Rey et al. [8] in Venezuela suggest that there is a potential relationship between the properties of the soil that generates a stress situation in the plant caused by specific abiotic factors, which next would enhance the deleterious effect of certain microorganisms, such as fungi and bacteria (fungal–bacterial complex), inducing the expression of wilt symptoms in the plant. Thus, according to Rey et al. [8], BW is associated with a fungal–bacterial complex, with some agroecological conditions characterized by silty soils presenting drainage problems and with nutritional imbalances, typical of the lacustrine soils that are accentuated by inappropriate fertilization regimens in the last few years. Additionally, the appearance of, and increase in, the disease is associated with an average annual decrease in precipitation and an increase in maximum temperatures [9]. 

Despite the technological advances, it is difficult to find studies that relate soil properties to disease incidence through the use of supervised methods, such as Random Forest (RF), Orthogonal Least Squares of Discriminant Analysis (OPLS-DA) and other algorithms. RF is a supervised learning classifier that can be used in complex situations [10,11] and has been proved to be a highly accurate classifier, but it has rarely been applied in the identification of soil properties associated with the incidence of diseases, such as BW [12,13].

In order to anticipate the potential occurrence of BW disease, it would be very valuable if certain soil characteristics could be associated with a major risk of the occurrence of BW. This research presents a study aimed to validate the hypothesis that it is possible to identify the specific soil properties associated with a high incidence of BW, using supervised methods such as RF and OPLS-DA, whose results can be of straightforward agronomic and environmental interpretation.

## 2. Results

### 2.1. Incidence of BW in Experimental Lots

The analysis of the identification of pathogenic microorganisms revealed the presence of bacteria (*Pectobacterium* and *Erwinia* genera) and fungi (*F. moniliforme*, *F. oxysporum*, and *F. solani*). These microorganisms were also found by Sabadell [14] in tissues with BW symptoms from the Canary Islands (Spain), and recently by Rey et al. [8] in the lacustrine banana soils of Venezuela, but no vascular *Fusarium oxysporum* isolates were recovered from the internal plant vascular tissue, which indicated that the symptoms observed in the field plot were associated with BW and not with Fusarium wilt.

**Figure 1:**
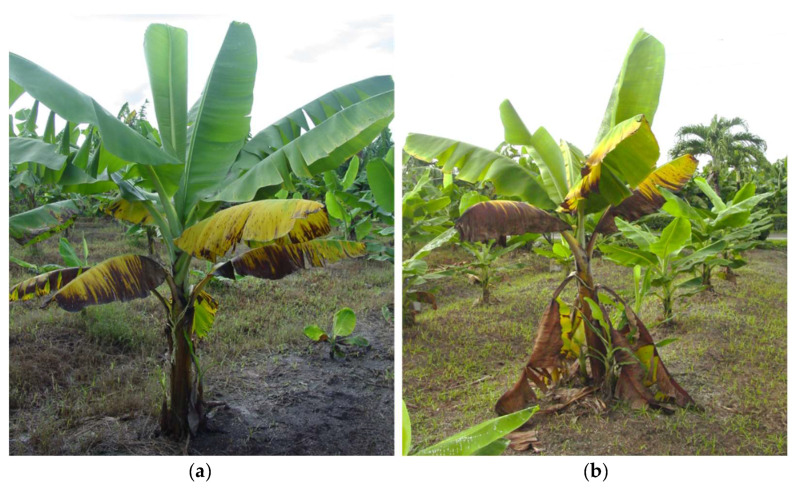
Symptoms of Banana Wilt disease in study area. (**a**) The yellow margins on the leaves and the necrotic stripes surrounded by the yellow margins on the lower or older leaves; (**b**) Set of dead leaves hanging from the pseudostem of a plant affected with Banana Wilt disease.

The symptoms of BW disease are shown in Figure 1. Generally, yellowing begins on the lower or older leaves. The margin of each leaf turns pale green to yellow, necrotic stripes appear surrounded by a yellow margin, and the leaf eventually dies (Figure 1a). The lower leaves die and hang from the pseudostem, resembling a skirt (Figure 1b). According to Beer et al. [15], the base of the leaf remains green and healthy, while its distal part dies. Often one to four upper leaves remain green, but are smaller in size and their development stops. New leaf growth can occur, but the bunches in this case are generally small with short and thin bananas, which generates economic losses due to the rejection of the fruit in the market.

**Figure 2:**
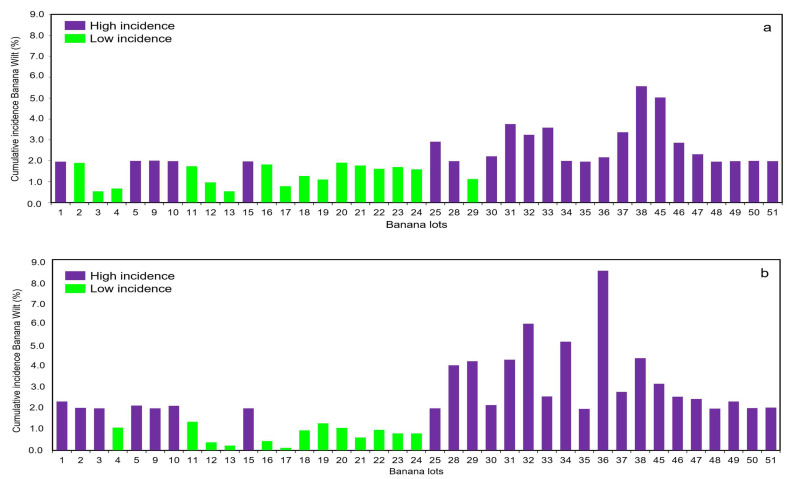
Cumulative incidence (%) of Banana Wilt in the study area during 2016 (**a**) and 2017 (**b**) (*n* = 78; mean = 2.17 ± 1.40%; min = 0.11%; max = 8.47%; asymmetry = 1.78; kurtosis = 4.46; P_50_ = 1.90%).

All of the lots evaluated (*n* = 78) in the study area have BW disease. The percentage of the lots with a low incidence (<1.90%) of BW reached 37.18% (*n* = 29), while the lots with a high incidence (≥1.90%) represented 62.82% (*n* = 49) (Figure 2). The highest incidence values were found in lot 36 with 8.47%, lot 32 (5.97%) and lot 34 (5.13%) for the year 2017 (Figure 2b), while during 2016 the maximum incidence values were registered in lots 38 and 45 with 5.57% and 5.03%, respectively. On the other hand, lots 12, 13 and lot 17 presented low incidence values that did not exceed 1.0% in both of the years of evaluation. For the entire dataset, the mean incidence was 2.17 ± 1.40 with a P_50_ of 1.90 (Figure 2a). However, there were no significant differences in the BW incidence according to the date on which different banana lots were established within the study area, according to the Kruskal–Wallis test (*p* value: 0.107). 

### 2.2. Description of Soil Properties in Experimental Lots

**Figure 3:**
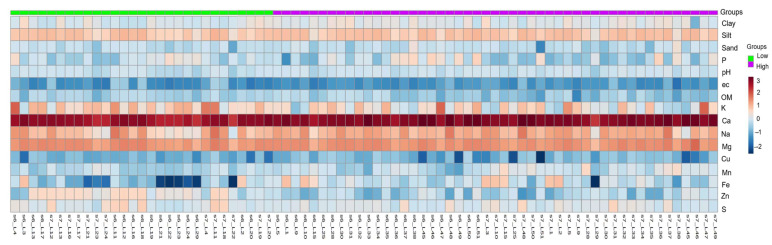
Heatmap generated from soil data of the banana lots with low (green) or high (purple) incidence of BW evaluated in year 2016 (s6) and year 2017 (s7), which represents increasing concentration values of the soil variables (blue to red color) for the study periods.

Figure 3 shows the results of the heat map of the soil data, classified into the high and low incidence groups. The heat map provides an intuitive visualization of the data used; each colored cell in the map corresponds to a concentration value in the data table, with the soil properties in the rows and the 78 banana lots in the columns. In general, the soils with a high incidence of BW presented with loam to silty loam textures, with a predominance of the particles with an equivalent diameter between 2 and 50 µm. In these soils, the banana lots classified as a high incidence of BW showed high values of Na, Fe and Mg, with slightly higher pH values (Figure 3).

On the other hand, the characteristics of the parental material of these soils produced very high levels of Ca. The limitations for the development of the roots in these soils with a high incidence of BW could be associated with chemical conditions, such as the presence of a high CaCO_3_ content, the limiting ratios being Ca Mg^−1^ and Ca K^−1^ (data not shown). The sodium levels were high in most of the lots with a high incidence of BW, which could generate toxicity problems for the plants and low structural stability in the soils. Likewise, low levels of Cu were observed in the lots with a low incidence of BW. The metabolic nature of these elements means that their deficiency can greatly affect the development of the crop. It is important to highlight that in some of the lots with a high incidence of BW, high levels of P were present on the surface, possibly due to overfertilization. 

In the very loamy soils, with low permeability and limited drainage, and with a nutrient imbalance, BW disease was more frequent. Additionally, in the soils showing a high incidence of BW, the clay content was slightly higher, whereas the K and Zn contents was slightly lower. However, a high incidence of BW occurred in those plant lots where the Ca content was higher, while the soils were more saline in depth.

### 2.3. Wilcoxon Rank Test 

**Figure 4:**
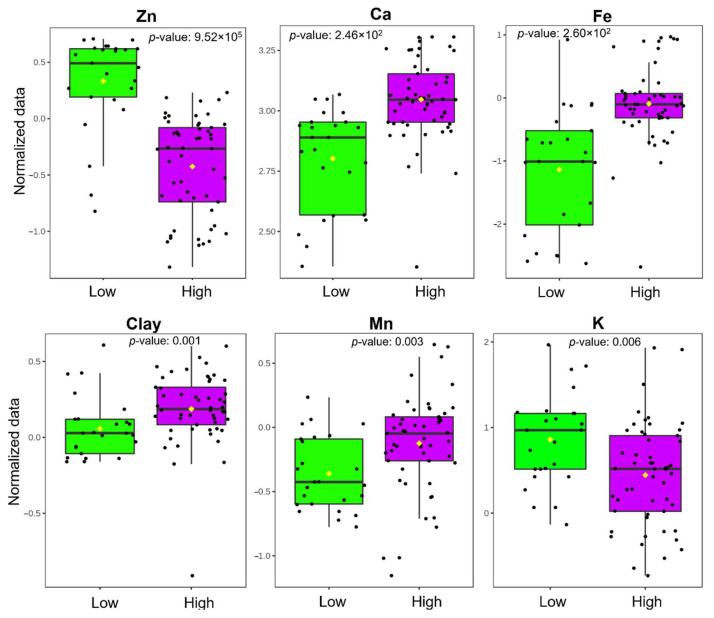
Box plots of levels of soil variables showing significant differences between low and high BW incidence based on the Wilcoxon’s test.

For a direct comparison of the soil variables’ levels, the Wilcoxon analysis was used to identify the critical significant variables differentiating between the groups with a low and high incidence of BW. The analysis revealed a total of six significant soil variables (adjusted *p* value < 0.05) (Table 1): Zn, Ca, Fe, Clay, Mn and K. In our study, a small fraction of false positives could be accepted as substantially increasing the total number of discoveries; therefore, the false discovery rate (FDR) obtained is usually appropriate and useful. The FDR is the rate at which the so-called significant features are actually null. The significant and most important soil variables that were responsible for the observed differentiation between the two BW incidence groups are shown in Figure 4.

### 2.4. Identification of Important Soil Variables

**Figure 5:**
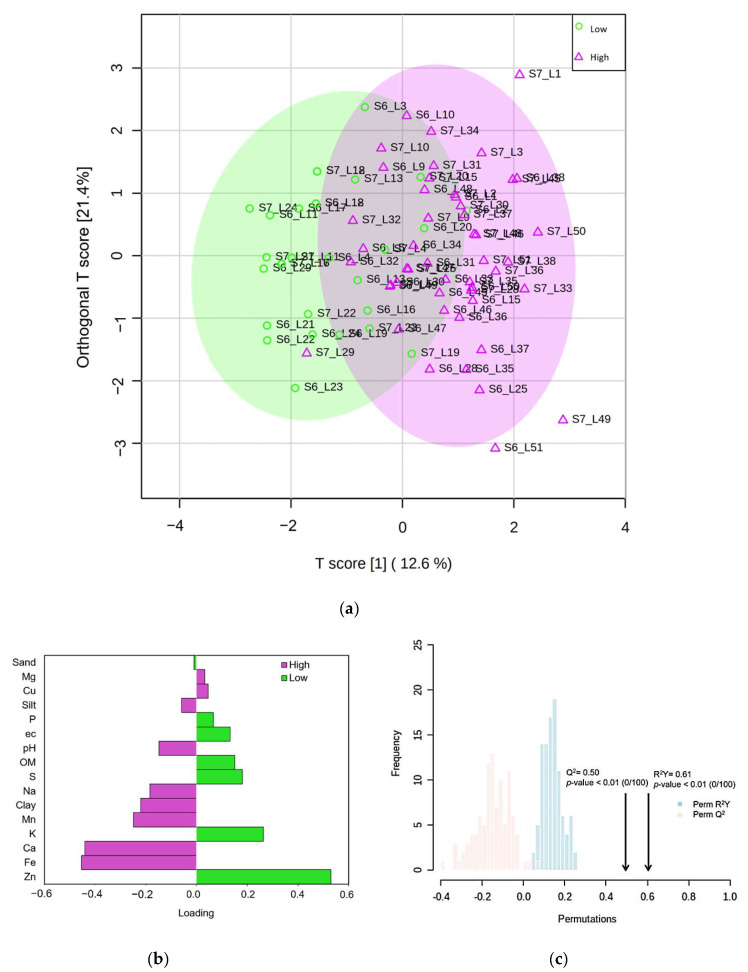
(**a**) OPLS-DA score plot of all soil variables, based separation of the incidence (low incidence of BW, *n* = 29; high incidence of BW, *n* = 49); (**b**) Loading plot weights of each variable selected from OPLS-DA; The color indicates the class in which the variable has the maximum level of expression; (**c**) internal validation of the corresponding OPLS-DA model by permutation analysis (*n* = 100); fraction of the variance of descriptor class response (Y) (R^2^Y) = 0.61 (blue bars), *p* value < 0.01; fraction of the variance predicted (cross-validated) (Q^2^) = 0.50 (red bars), *p* value < 0.01.

The results of the descriptive analysis (Table 2) indicated important differences between the characteristics of the soils of the sampled banana lots. The variable importance in the projection (VIP) values were obtained from the OPLS-DA model. The VIP was taken for selection, and those variables with a VIP > 1 were considered as possible candidate variables for the group discrimination (Table 2). Accordingly, the analysis revealed prominent values in three variables: K, Fe and Zn. On the other hand, as shown in Figure 5a, the OPLS-DA allowed us to analyze the information collected in the predictive component independently from the orthogonal components. That is, it allowed the separation of the variability responsible for the discrimination from the noise generated by the uncorrelated variability. For this reason, the OPLS-DA was the method chosen for the selection of the relevant variables in the discrimination of groups. In addition, based on the loading values > 0.2, the OPLS-DA identified six critical variables: Clay, Mn, K, Ca, Fe and Zn (Figure 5b). Besides, the OPLS-DA model showed a proper fitting of the data (R^2^Y = 0.61, *p* value < 0.01), and exhibited good predictive power (Q^2^ = 0.50, *p* value < 0.01) (Figure 5c).

### 2.5. Classifier Performance and Accuracy Assessment

**Figure 6:**
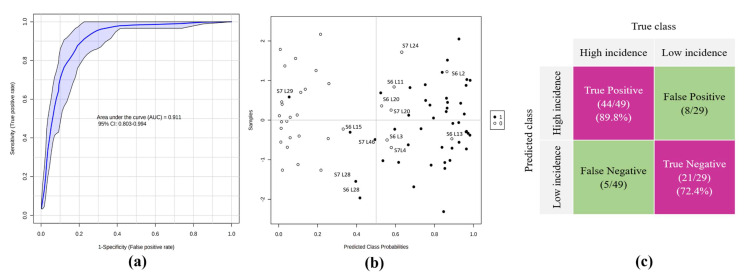
Classification of bananas lots according to the incidence of banana wilt (BW). (**a**) Receiver operating characteristic (ROC) curve after obtained by Random Forest as classification method. The values generated for the area under the curve (AUC) (0.91) along with the 95% confidence intervals (CI) (0.80–0.99) are given within the graph and accuracy: 84.10%; (**b**) Predicted class probabilities for each banana lot, allowing display of misclassified bananas lots (lots of high BW incidence are shown as black dots; lots of low BW are shown as white dots). Since a balanced subsampling approach is used for model training, the classification limit is always in the center (x = 0.5, the dotted line); (**c**) Confusion matrix showing the number of true positives (44/49), true negatives (21/29), false positives (8/29) and false negatives (5/49). Sensitivity and specificity are given in the regions highlighted in purple, being 89.80% and 72.40%, respectively.

Table 3 shows the measures of the importance of the soil variables selected by the RF model. The results establish the frequency with which an independent variable is selected greater than/equal to a defined importance threshold (0.5). The Mean Decrease Accuracy (MDA) allows for the visualization of the relative impact on the performance of the RF classifier by subtracting each specific soil variable. Figure 6 shows the classification results after the RF analysis; the receiver operating characteristic (ROC) curve of the best-performing model indicated an area under the curve (AUC) of 0.91 (95% confidence interval CI: 0.80% to 0.99%) (Figure 6a). The scores plot (Figure 6b) shows the predicted class probabilities for all of the samples included in the analysis, indicating the correct classification of 44 banana lots out of 49 with a high incidence of BW, and 21 banana lots out of 29 with a low incidence. 

Our results showed the great power of the RF classifier to correctly differentiate the lots of bananas with a high or low BW incidence. Furthermore, our proposed system reached 89.80% sensitivity and 72.40% specificity in the test dataset, which implies that most of the banana lots with a low BW incidence were correctly classified with a false negative (FN) rate of 5/49, and most of the banana lots with a high BW incidence were also correctly classified with a false positive (FP) rate of 8/29 (Figure 6c).

Finally, the McNemar test was used to determine if the observed vs. predictive proportions of the banana lots with a high and low incidence of BW were different. The results establish that the *p* value of the McNemar test (0.41) is greater than 0.05, so there is no evidence to reject the null hypothesis, and it is concluded that there are no significant differences in the proportion of banana lots with a high and low incidence of BW before (observed data) and after classification with RF (predictive data).

## 3. Discussion

Banana Wilt is a disease of unknown etiology that has not yet been properly studied. Indeed, the incidence of BW has only been assessed in a few countries, including Costa Rica, where a BW incidence of 7.3% was reported [16]; in Colombia, where an incidence of 0.31% was reported in some of the banana-producing areas with a prevalence of 4.30% [17]; and in Indonesia, where the average incidence of BW in 15 provinces was as high as 24% [18]. 

In the case of the banana areas located in the Aragua state of Venezuela, Martínez et al. [19], Ramírez et al. [20] and Rey et al. [8] reported incidences of BW ranging from 0.32% to 11.41% in different plant lots. These values are similar to those obtained in our study, where the vast majority of the foci showing an incidence of BW were centralized between lots 31 to 46 of the farm sampled and for both of the years evaluated. This could suggest that the spread of the disease may be linked to specific soil physical–chemical characteristics, combined in some degree with poor agronomic management (inappropriate fertilization) that generates a significant nutritional imbalance in the soil. 

The identification of the symptoms associated with BW represented the first step in understanding and identifying the causes of the disease in the field and distinguishing the areas affected by the disease, to later perform a classification based on certain previously established statistical, economic and agronomic management parameters. In our study, we established two levels (low and high) for describing the incidence of BW, based on previous experience in the banana field plots in Venezuela presenting similar type of soils and agronomical practices (J. C. Rey, personal communication). This threshold incidence value of 1.90% was selected as that inducing severe yield loss. 

The studies indicated that the soil factors, specifically its physical and chemical properties, are closely associated with the occurrence of BW in bananas [7,8,14,15]. In the present study, using a RF model, we identified soil differences in six soil variables (i.e., Zn, Fe, Ca, K, Mn and Clay) between the zones with different levels of BW incidence. The K contents were highest (5.6–984.0 mg kg^−1^) in the group of lots with a low incidence of BW. However, Ca contents were excessively high in both of the groups, with the concentrations being more notable in the lots with a high incidence of BW (6472–16,648 mg kg^−1^), due to the lacustrine origin of the soils, which can generate K and Mg deficiencies in the plants. In relation to the microelements, Fe (0.06–78.40 mg kg^−1^) and Mn (0.8–58.4 mg kg^−1^) were present at high levels in the group of lots with a high incidence of BW, while Zn was at low levels (0.3–30.4 mg kg^−1^) (Table 3). These high Fe and Mn contents could be associated with a higher clay content that can generate drainage problems. Under these conditions of excess humidity, the solubility of Fe^2+^ and Mn^2+^ increases [21].

Regarding Zn, in the Canary Islands, the authors of [22] demonstrated that the application of Zn in the soil notably reduced the incidence and severity of BW because this type of soil shows a Zn deficiency. Therefore, in our study, conducted in the soils of Aragua, Venezuela, the low levels of this element in the plant lots with a high incidence of BW may have favored the appearance of the BW symptoms. According to Domínguez et al. [4], the banana soils in the Canary Islands that presented severe BW problems showed a tendency to the formation of stable aggregates of clays, that with an excess of irrigation favored anaerobiosis in the soil and high concentrations of Fe, which caused compaction when the soil became dry. These relationships of the clay content (1–40%) with the water and the detrimental effect of compaction in banana soils results in a decrease in the productivity and plant height, and a reduction in the number of offspring plants in the banana production unit. Additionally, according to the results of Dorel [23] and Sabadell [14] the most significant effect would be related to the reduction in the absorption of N, P, K, Ca and the massive absorption of Mn.

The results of our analysis established that the heavy texture in the lots with a high incidence of BW favored the appearance of symptoms, agreeing with the other studies that found that this disease developed in the presence of soils with a heavy texture [24] and poor drainage [25], in conditions of high humidity, favoring infection by deleterious microorganisms in the lateral rootlets.

The study by Rey et al. [8] establishes that the variables that showed the highest significant correlation with the incidence of BW were the sand and silt content, organic carbon, exchangeable Mg content and the Ca/Mg ratio. The authors found that a positive correlation was observed with BW incidence for the silt content and the Ca and Mg levels in the banana soils of Aragua, indicating that in very silty soils with low permeability and limited drainage, it was more frequent to find a high incidence of BW. Likewise, they found that, the C/N ratio and the K content, the nutritional relationships between the exchangeable cations (Ca, Mg and K) and the Zn content were the variables that had the greatest importance in the differentiation between the field areas, coinciding with the results of this study.

Our results also showed that the incidence of the disease was not uniform throughout the farm; the most affected areas had very silty soils with drainage problems, certain nutrient deficiencies and nutritional imbalances, related to the natural condition of the lacustrine soils and, surely, the lack of appropriate fertilization cycles in recent years [8]. 

In recent times, modern approaches, such as machine learning and deep learning algorithms, have been employed to identify the characteristics of banana agroecosystems that could be affecting productivity and the appearance of diseases in the field. Several investigations were carried out in the field of machine learning for the detection and diagnosis of banana diseases, using RF [11,12,26,27,28], artificial neural networks [11], support vector machine (SVM) [10,11,29,30] and decision trees [26], among others. This study aimed to use a RF model analysis strategy to determine the soil variables that could favor the development of BW disease, with the final aim of helping to avoid using those soils or promoting the application of the appropriate corrective fertilization treatments.

In those studies, reported above, the machine learning analysis approaches were used to detect Fusarium wilt and Black Sigatoka diseases using aerial images, but none of them used in situ soil data to predict the occurrence of a banana disease, as is the case in our study. This evidences the existence of an information gap regarding the application of these novel algorithm-based techniques, using data from the sampled soils. Our study is a pioneer in showing results from the application of supervised methods, such as OPLS-DA and the RF algorithm, to identify the soil variables associated with BW incidence. According to our results, it is reported for the first time that soil variables, such as Zn, Fe, Ca, K, Mn and Clay content, could be promising new soil indicators to classify the lots of bananas prone to show a higher incidence of BW disease in the lacustrine soils in Venezuela.

The RF classifier achieved a significant advantage over the classifiers used in previous works [11,12,28]. The characteristics of the RF classifier, and the way in which the most important soil variables are selected through the OPLS-DA, determine the performance of the RF classifier. However, the precision of classifying the banana lots with different levels of BW incidence can be affected by many different factors, such as the quality and representativeness of the information obtained, the performance of the characteristic extraction algorithm, and the subsets used for training and testing purposes, as established by the studies of [11,12]. The results of our study showed that RF performed well in differentiating the banana lots with a high or low BW incidence. More interestingly, our model provides an easy, fast and inexpensive method to accurately identify the risk of incidence of BW in bananas. 

Nevertheless, we are aware that it is not only the soil properties that may be directly related to the plants that develop BW, since it is a disease caused by a fungal–bacterial complex. Consequently, it is logical to think that the climatic variables of the site, other than the physical and biological soil properties, and the physiological and agronomic management of the plantation, among other factors, could also have an important influence on the manifestation of the disease. However, all of those factors were not the object of this study; so, it would be necessary to establish additional methods of analysis that would allow for the analysis of the complexity of this type of disease, to obtain findings that do not depend on a single method of analysis and to explore other potential factors that may influence the development of BW.

## 4. Materials and Methods

### 4.1. Study Area

**Figure 7:**
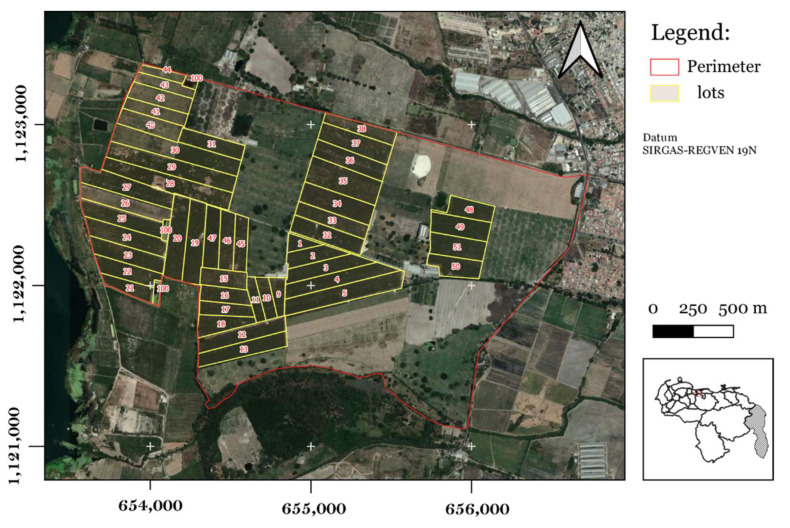
Geographical location of the study area with banana lots (marked with yellow color boundaries).

The study was carried out in a banana plantation located in the Aragua state, with 205 ha planted with Cavendish cv. Pineo Gigante (67.58° W, 10.14° N; Figure 7). These plants had at the time of sampling: (i) a leaf number from 16 to 18; (ii) height values ranging from 3.5 to 4.5 m; and (iii) a growth period from 9 to 10 months. This region is characterized by a Tropical Savanna climate (Aw). The annual mean rainfall is 980 mm [31] and shows a marked seasonal pattern, with a wet season from May to October. The mean annual temperature is 26.2 °C, whereas the mean annual relative humidity is 70.0% [32]. The terrain relief is mostly flat (slope ranging 0–2%). The predominant types of soil are Mollisol and Entisol, which are mostly of lacustrine origin, with medium textures, high nutrient availability, moderate to good drainage, soil pH varying from neutral to alkaline, good fertility and high soil organic matter content [33,34].

### 4.2. Soil Sampling

A systematic soil sampling was carried out in 39 banana lots sampled during January 2016 and 2017 (total banana lots sampled, *n* = 78) (Figure 7). These lots were established at different periods at the time of disease monitoring (<6 years, 6 to 12 years, and >12 years) [8]. The sampling was conducted following the guidelines of Lozano et al. [35], with an approximate distance of 150 m between the sampling sites. The composite soil samples were obtained in each of the banana lots, in the first horizon at a depth of 0 to 20.0 ± 5.0 cm. The samples were subjected to soil analysis for fertility characterization purposes; in total, 16 soil variables were determined including: percentage of sand, silt and clay [36]; soil reaction (pH); electrical conductivity (EC, dS m^−1^) in suspension 1: 2 (soil: water) [37]; organic matter (OM, %) [38]; available contents of potassium (K, mg kg^−1^); sodium (Na, mg kg^−1^); magnesium (Mg, mg kg^−1^); calcium (Ca, mg kg^−1^); manganese (Mn, mg kg^−1^); iron (Fe, mg kg^−1^); zinc (Zn, mg kg^−1^); copper (Cu, mg kg^−1^); sulfur (S, mg kg^−1^) and phosphorus (P, mg kg^−1^) [39]. 

### 4.3. Banana Wilt Incidence

Before the beginning of the study, the plants with the typical symptoms of BW disease were located and identified in all of the lots of the farm, from which the tissue samples were taken from the pseudostem and roots, for the identification of the pathogenic microorganisms. The isolation method, in PDA culture medium and humid chamber, was used, in the laboratory of the Faculty of Agronomy of the Central University of Venezuela. 

For the identification of the BW incidence in the field, in each banana lot each banana plant was individually inspected on a monthly basis for the presence of symptoms compatible with BW. The banana plants showing BW symptoms were eliminated in each lot and each evaluation period. Therefore, in the next monthly inspection, only the number of plants with new BW symptoms to that date were counted. The cumulative incidence of BW was determined in each of the 78 banana lots sampled during 2016 and 2017, using the guidelines by Bosman [40]. The main aim of the continuous monitoring of BW incidence was to determine the new cases of BW that occurred in the total population of plants in each banana lot in a given plot and sampling time and for all of the physiological plant stages growing simultaneously. The harvest of the fruit was carried out throughout the year, which is interpreted as a staggered harvest, so that in the same lot it is possible that the plants are in different phenological phases: Vegetative; Floral and Fruiting; that is why the annual accumulated incidence was obtained to prevent the incidence of BW from being confused with plant age. Within a banana lot, a plant grows for a maximum of 11- to 12-month period when the fruit is harvested and the mother plant removed. Hence, the cumulative incidence rate is calculated as the sum of the monthly incidence of BW values of all of the plants at different phenological stages in percentage for each banana lot in a particular year according to Equation (1):

(1) $$\mathrm{Cumulative}\;\mathrm{incidence}\;\mathrm{rate}\;(\%)=100\;\times \;\overset{12}{\underset{i=1}{\sum }}\frac{n{}^{\circ}\;\mathrm{of}\;\mathrm{diseased}\;\mathrm{plants}\;(\mathrm{BW})}{\mathrm{Total}\;\mathrm{plants}\;\mathrm{planted}}$$

In the scientific literature, there is no information describing the threshold values to establish the categories for BW incidence for the study area, nor in any other banana areas of Venezuela. The percentiles were established in agronomy as an important alternative to disease incidence indicators in bananas [41,42]. In this sense, the percentile (50) (P_50_) or median represented by the value below which a certain proportion of the observations falls was selected. In this study, the P_50_ (and thus also the percentile rank classes) offer an alternative to the mean-based ratios for the disease incidence classes. The selection of this measure of the statistical position is based on the low influence of the extreme values of the distribution, such as the mean value; as additionally, the non-dependence of the choice of the specific probability density functions compared to the arithmetic mean, which requires normally distributed data [43].

The two percentile-rank classes are aggregated as follows: low incidence of BW < 1.90% (incidence values of BW with a percentile less than the P_50_); and high incidence of BW ≥ 1.90% (incidence values of BW with a higher percentile equal to the P_50_).This high incidence value would represent a decrease of up to 13,300 kg ha^−1^ year^−1^ in those banana lots showing an incidence of BW of 1.90% and was selected based on the information provided by J.C. Rey (personal communication, 28 September 2019) and several years of experience observing yield losses associated with BW.

### 4.4. Data Analysis

**Figure 8:**
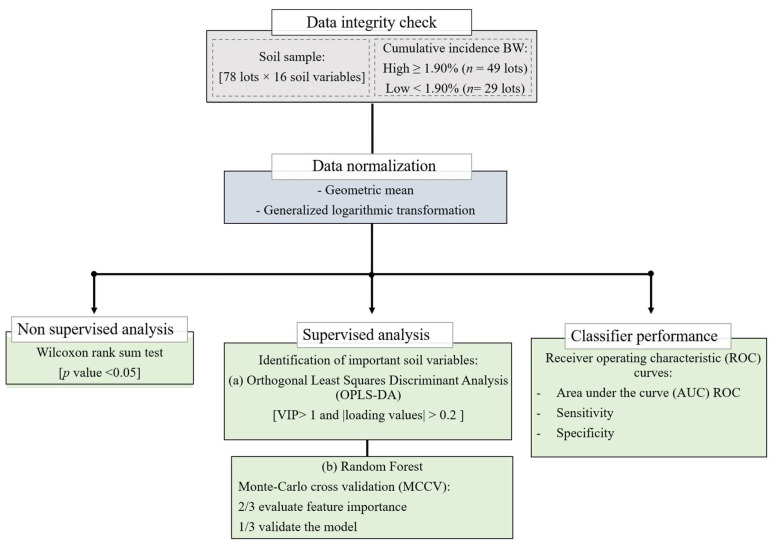
General scheme of the data analysis procedures (sample size, *n* = 78; variable size, *n* = 16) using non supervised and supervised analysis.

Before the data analysis, we checked the data integrity. The normalization of the soil variables was carried out using the statistical package in R software version 4.0.2 (R Core Team, Austria) [44] based on the geometric mean, and a generalized logarithmic transformation using “glog” function in R was performed to make the variables comparable among themselves due to differences in the units to measure them [45,46]. Figure 8 shows the general scheme of the data analysis procedures followed in this work.

#### 4.4.1. Identification of Important Soil Variables 

For the identification of the relevant soil variables characterizing the incidence of BW, a Wilcoxon rank sum test was performed to find the most important features of the soil variables at a threshold *p* value < 0.05 [45], showing the differences between the group of bananas lots with a low and high incidence of BW. Next, an Orthogonal Least Squares Discriminant Analysis (OPLS-DA) was used to reduce the number of the soil variables in the high-dimensional data to produce a robust and easy-to-interpret model, and to identify the main soil characteristics that drive the separation of the plant lots based on BW incidence (low or high). This multivariate statistical analysis was carried out using “ropls” R packages [47].

The variable importance in projection (VIP) > 1, and the corresponding |loading values| > 0.2 in the model were used to identify the variables responsible for distinguishing both of the BW categories [48]. Furthermore, a permutation test with 100 permutations was employed to validate the performance of OPLS-DA model. For the quality criteria, we chose in the OPLS-DA model, the R^2^Y (goodness of fit parameter) and Q^2^ (predictive ability parameter) > 0.5 [49].

#### 4.4.2. Classifier Performance and Accuracy Assessment

The random forest (RF) algorithm was used as a machine-learning approach for classifying the lots with a high and low incidence of BW [50]. The RF models allow for the prediction of unknown samples (i.e., a test dataset) after training on a known dataset (i.e., a training dataset). The receiver operating characteristic (ROC) curves were generated by Monte Carlo cross validation (MCCV) [51], that is, a cross validation approach which creates multiple random splits of the dataset into training and validation data. In each MCCV, two/three of the samples were used to evaluate the feature importance, and the remaining third were used to validate the model created in the first step [52,53].

To determine the predictive performance of the model, the graphs of the ROC curve were used, from which the sensitivity was defined as the relationship between the number of P correctly classified and the total P observed, against “1—specificity” (specificity is the relationship between the number of N correctly classified and the total N observed). A model will have a high predictive performance if at low values of “1—specificity” a high sensitivity is obtained, that is, a good capacity to correctly classify P with a low number of false positives. This yields a curve closer to the upper left corner [54]. The Area under the ROC curve (AUC) quantifies this relationship, so that a model is considered acceptable if the AUC ≥ 0.7, excellent if the AUC ≥ 0.8 and outstanding if the AUC ≥ 0.9. 

## 5. Conclusions

This study was focused on an analysis of the key soil properties that play an important role in the incidence of BW. So far, crop-disease detection models primarily focus on leaf symptoms through image recognition technology. This means that the diseases can be detected only after they have appeared. In the present study, by using a random forest analysis approach, we identified that the risk of low or high incidence of BW in a banana farm in Venezuela could be associated with the differences in six key soil variables, including Zn, Fe, K, Ca, Mn and Clay content. The findings may contribute to increasing our understanding of the basic mechanisms and progression of BW incidence, and indicated that these soil variables are potentially the determining factors of a risk of high BW incidence in the tropical lacustrine soils of Venezuela.

Although the Random Forest analysis performed well in this particular study, and its performance in other banana areas in Venezuela has not yet been proven, we consider that this machine learning algorithm, using the soil properties as indicators, has the potential to be further explored as a simple and effective tool in banana areas with the risk of developing BW.

Our results open the field for further research in which we could quantitatively predict the risk of BW in banana fields based on available, or relatively easy to gather, information, which in turn could allow farm managers to implement preventive measures to minimize BW risk and target other techniques (e.g., plant sampling, withdrawal of infested material) on the areas where there is maximum risk. 

In the future, new research can be improved through the systematic use of new locations to obtain a much larger database of BW-affected plants, and also to take into consideration various environmental, physiological and agronomic variables, among others, and apply new and different statistical analyses that may help to identify other factors potentially associated with BW development.

## Figures and Tables

**Table 1:** Important variables selected by Wilcoxon Rank Test with threshold 0.05.

Variable	V	*p* Value	−log10(*p*)	FDR
Zn	1199	9.52 × 10^5^	80.21	1.52 × 10^3^
Ca	222	2.46 × 10^2^	56.08	1.38 × 10^1^
Fe	223	2.60 × 10^2^	55.85	1.38 × 10^1^
Clay	357	0.001	29.61	0.004
Mn	386	0.003	25.05	0.009
K	921	0.005	22.41	0.015

Note: (V): The V-statistic. These values are based on the unpaired data; FDR: The false discovery rate.

**Table 2:** Input variables used in model’s construction (mean ± standard deviation, coefficient of variation, maximum and minimum) and the variable importance in the projection (VIP) values obtained from the OPLS-DA model.

Variable	Mean ± SD	Median	CV (%)	Min	Max	VIP
Clay (%)	16.10 ± 7.86	15.00	48.78	1.00	40.00	0.36
Silt (%)	76.82 ± 9.75	78.30	12.70	39.93	90.84	0.08
Sand (%)	7.08 ± 4.98	6.27	70.33	0.38	38.07	0.01
pH	7.86 ± 0.22	7.85	2.74	7.41	8.55	0.15
EC (dS m^−1^)	0.65 ± 0.53	0.45	82.51	0.21	2.58	0.28
OM (%)	3.39 ± 1.52	3.50	44.86	0.25	6.33	0.32
P (mg kg^−1^)	12.98 ± 15.48	5.67	119.32	0.35	54.97	0.24
K (mg kg^−1^)	110.57 ± 229.26	35.60	207.34	1.48	1336.00	1.16
Ca (mg kg^−1^)	9704.51 ± 2968.46	8892.00	30.59	4936.00	16,648.00	0.68
Na (mg kg^−1^)	152.80 ± 97.07	132.40	62.33	10.72	472.00	0.55
Mg (mg kg^−1^)	300.47 ± 55.34	296.00	18.65	216.00	640.00	0.06
Cu (mg kg^−1^)	1.41 ± 0.87	1.60	61.20	0.03	3.20	0.15
Mn (mg kg^−1^)	9.47 ± 10.81	5.60	114.12	0.80	58.40	0.66
Fe (mg kg^−1^)	13.39 ± 22.59	5.20	168.72	0.04	78.40	2.91
Zn (mg kg^−1^)	13.21 ± 13.28	7.60	100.57	0.36	36.80	2.11
S (mg kg^−1^)	17.09 ± 11.86	11.84	69.43	6.47	48.80	0.34

**Table 3:** Frequencies of variables being selected (%), Mean Decrease Accuracy and descriptive statistics of the model soil variables with Random Forest (Accuracy: 84.10%).

Variables	Frequencies of Being Selected (%)	Mean Decrease Accuracy	Low Incidence Group (*n* = 29)		High Incidence Group (*n* = 49)	
			Mean ± SD	Range	Mean ± SD	Range
Zn (mg kg^−1^)	1.00	0.18	26.03 ± 12.98	(1.60–38.00)	5.66 ± 5.41	(0.36–30.40)
Fe (mg kg^−1^)	1.00	0.05	7.04 ± 16.84	(0.04–69.60)	17.14 ± 24.79	(0.06–78.40)
Ca (mg kg^−1^)	0.97	0.04	7326.34 ± 1675.99	(4936.00–11,496.00)	11,111.18 ± 2657.83	(6472.0–16,648.0)
Clay (%)	0.88	0.01	15.66 ± 8.70	(5.00–31.00)	16.37 ± 7.39	(1.00–40.00)
K (mg kg^−1^)	0.65	0.01	142.20 ± 199.53	(5.60–984.00)	91.83 ± 245.22	(1.50–1336.0)
Mn (mg kg^−1^)	0.50	0.01	6.38 ± 6.19	(1.60–33.60)	11.30 ± 12.49	(0.80–58.40)

## Data Availability

Not applicable.
